# Adsorption of Milk Proteins (*β*-Casein and *β*-Lactoglobulin) and BSA onto Hydrophobic Surfaces

**DOI:** 10.3390/ma10080893

**Published:** 2017-08-02

**Authors:** Leonor Pérez-Fuentes, Carlos Drummond, Jordi Faraudo, Delfi Bastos-González

**Affiliations:** 1 Biocolloid and Fluid Physics Group, Department of Applied Physics, University of Granada, Av. Fuentenueva 2, E-18001 Granada, Spain; lpfuentes@ugr.es (L.P.-F.); dbastos@ugr.es (D.B.-G.); 2 CNRS, Centre de Recherche Paul Pascal (CRPP), UPR 8641, F3300 Pessac, France; drummond@crpp-bordeaux.cnrs.fr; 3 Université de Bordeaux, CRPP, UPR 8641, F-33600 Pessac, France; 4 Institut de Ciència de Materials de Barcelona (ICMAB-CSIC), Campus de la UAB, E-08193 Bellaterra, Barcelona, Spain

**Keywords:** proteins, QCM, MD simulations, hydrophobic effect, ion condensation, electrokinetic mobility

## Abstract

Here, we study films of proteins over planar surfaces and protein-coated microspheres obtained from the adsorption of three different proteins (β-casein, β-lactoglobulin and bovine serum albumin (BSA)). The investigation of protein films in planar surfaces is performed by combining quartz crystal microbalance (QCM) and atomic force microscopy (AFM) measurements with all-atomic molecular dynamics (MD) simulations. We found that BSA and β-lactoglobulin form compact monolayers, almost without interstices between the proteins. However, β-casein adsorbs forming multilayers. The study of the electrokinetic mobility of protein-coated latex microspheres shows substantial condensation of ions from the buffer over the complexes, as predicted from ion condensation theories. The electrokinetic behavior of the latex-protein complexes is dominated by the charge of the proteins and the phenomenon of ion condensation, whereas the charge of the latex colloids plays only a minor role.

## 1. Introduction

Protein adsorption is a basic step in many natural processes and biomedical applications such as drug delivery [1] or disease detection [2,3]. Enhancement [4,5] or suppression [6,7,8] of protein film formation plays a basic role in biomedical implants. Due to its relevance, protein adsorption has been a broadly-studied phenomenon [8,9,10,11,12]. From the physico-chemical point of view, protein adsorption is far from trivial. A great variety of factors influence the adsorption process, either related to the surface (surface chemistry, charge and topography, etc.) or the protein (charge heterogeneity in the protein, possibility of conformational changes, etc.) [12,13]. Generally, proteins tend to adsorb both on hydrophobic and hydrophilic surfaces due to the variety of forces involved in the protein-surface interaction (e.g., solvation effects, hydrophobic effect, electrostatic interactions, etc.). However, the properties of a protein film (coverage, protein orientation and conformation, etc.) strongly depend on the nature of the interaction between the protein and the surface. This is due to the fact that proteins do not usually behave like rigid particles [12], and most proteins do not simply attach to or detach from a surface with certain adsorption and desorption probabilities. Instead, proteins are complex objects able to change conformation, deform, interact in different ways over the surface (aggregate or repel), change surface affinities during the adsorption, etc., so protein adsorption presents much greater complexity than the adsorption of simpler and smaller organic molecules that are usually considered rigid during adsorption [14].

A fundamental question in the field is therefore how to relate protein properties to the properties of the adsorbed protein film. In other words, the question is to develop a classification of proteins with respect to their adsorption behavior considering properties such as size, flexibility, structural stability and type of amino acids present at the protein surface. For example, small and rigid proteins like lysozyme or β-lactoglobulin are considered ‘hard’ proteins because they do not seem to experience significant structural alterations after adsorption onto surfaces [12,15]. Intermediate size proteins such as plasma proteins like albumins or immunoglobulins are reported to undergo conformational reorientations upon surface contact [12]. In any case, more extensive studies comparing the properties of proteins and the properties of the resulting films are needed to improve our understanding of the relationship between the properties of the proteins and of the adsorbed films. In this regard, it will be useful to combine experimental techniques for the characterization of the protein film with atomistic computer simulations, which will help to interpret mechanistically the obtained results and relate these to the molecular characteristics of the protein.

In this paper, we discuss this question by considering both theoretically- and experimentally- derived protein films made of two proteins with a similar number of amino acids and a similar size, but with significant structural differences. These proteins considered here will be β-casein and β-lactoglobulin. β-casein is a flexible protein that cannot be crystallized, so it is not possible to determine its structure by X-ray diffraction. Traditionally, it has been considered as a disordered protein with a random coil conformation in solution, but recent studies have challenged this view [16]. On the contrary, β-lactoglobulin is a globular protein with a secondary structure that corresponds predominantly to β-sheets [17]. The structure of this protein has been resolved with atomistic detail using X-ray diffraction [18]. As we said, β-lactoglobulin is considered a ‘hard’ protein. Both proteins are also interesting not only for the physico-chemical reasons mentioned above (similar size, but different secondary structure), but also for practical reasons: it is known that these are the major allergenic proteins of cow’s milk, especially in children [19,20]. From the perspective of materials science, these proteins are useful as building blocks for biopolymeric films in packaging as replacements for crude oil-based polymers [21]. Our previous work also shows that these proteins have different degrees of hydrophobicity and different interactions with organic hydrophobic ions [22]. In addition to these two proteins, we have also investigated films of bovine serum albumin (BSA). BSA is also a globular protein with a known X-ray structure, as β-lactoglobulin, but it has a much larger size. In this way, by comparing the three considered proteins, we can study both the effect of size and flexibility in the formation of protein films. BSA adsorption has been extensively studied in the literature both experimentally and by our own computer simulations [23,24,25,26,27] and will be useful as a reference for our studies with films of β-casein and β-lactoglobulin.

Hence, in this study, we have considered protein films of β-casein, β-lactoglobulin and BSA in order to compare films made of proteins with similar sizes and different secondary structures (β-casein vs. β-lactoglobulin) and also compare with those made of globular proteins with different sizes (β-lactoglobulin vs. BSA). Protein films adsorbed onto planar surfaces were studied experimentally using quartz crystal microbalance (QCM) and atomic force microscopy (AFM), and the results are interpreted with the help of atomistic molecular dynamics (MD) simulations. On the other hand, the electrical state of the adsorbed proteins is analyzed from the adsorption of the proteins onto latex particles. Electrokinetic measurements of the latex-protein complexes in buffered solutions as a function of pH are interpreted using theories of ion condensation.

## 2. Results and Discussion

### 2.1. MD Simulations of β-Casein

We first consider our results for β-casein in implicit solvent at neutral pH and 298 K without the adsorbing surface, and then, we will describe the results of MD simulations of adsorption onto a surface (see the Methods Section 3 for details). To the best of our knowledge, there are no previous published results for simulations of β-casein. The atomic coordinates of β-casein are not known experimentally, so we obtained the initial coordinates from an automated computational model [28] (see the Methods Section 3 for details).

**Figure 1:**
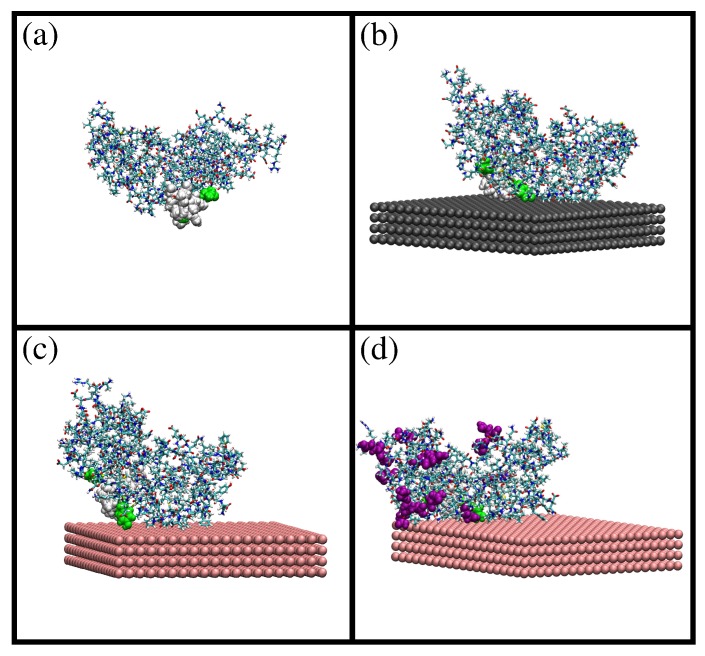
Snapshots from MD simulations of β-casein protein in different situations: (**a**) protein at pH 7 and 298 K; (**b**) protein at pH 7 and 298 K (charge −8e) adsorbed onto a neutral hydrophobic surface; (**c**) protein at pH 7 and 298 K (charge −8e) adsorbed onto a negatively-charged hydrophobic surface (σ = −0.62 e/nm^2^); and (**d**) protein at pH 4 and 298 K (charge +6e) adsorbed onto a negatively-charged hydrophobic surface (σ = −0.62 e/nm^2^). The residues 70, 73, 75–82, 85 and 126 (corresponding to the most hydrophobic region of the protein) are shown as van der Waals spheres. In (d), amino acids protonated due to the change in pH are shown as purple van der Waals spheres. These images were made with VMD [29].

A snapshot of the simulation results is shown in Figure 1a. The difference between the initial and the equilibrated atomic coordinates is measured with the root mean squared deviation (RMSD; see the Methods Section for details). We obtain an RMSD of ≈1 nm, which indicates a significant difference between equilibrated and initial coordinates. The solvent-accessible surface area (SASA) obtained at 298 K is larger than the SASA computed from the initial atomic coordinates (124 nm^2^ and 118 nm^2^, respectively). Concerning the size and shape of β-casein at 298 K, we obtained a radius of gyration of $R_{g}\approx$ 2.73 nm and an approximately spherical shape with small values of the asphericity Δ and shape *S* parameters ($\Delta \approx 6\times 10^{-2}$ and $S\approx -3\times 10^{-2}$). The obtained atomic coordinates from MD simulations were employed as the input configuration for a PropKasemi-empirical calculation of the dependence of the charge of the protein as a function of the pH isoelectric point. The obtained isoelectric point pI = 4.9 is in agreement with the pI ≈ 4.6–5.1 of the proteins employed in our experiments (see below and the Methods Section). The full curves of protein charge as a function of pH are given in the Supplementary Material (Figure S1).

The obtained configuration was employed as the initial condition for simulations of adsorption of a single β-casein onto a planar hydrophobic surface. We have considered three different cases: adsorption at pH 7 onto a neutral hydrophobic surface (Simulation 1), adsorption at pH 7 onto a negatively-charged surface with a charge density of −0.62 e/nm^2^ (Simulation 2) and the effect of the change of pH from 7–4 (Simulation 3). The effective charge of the protein is −8e at pH 7 and +6e at pH 4 (see the Supplementary Material).

Snapshots from these simulations are shown in Figure 1b–d). In all three cases, the protein is spontaneously adsorbed onto the surface. The value of SASA obtained after adsorption is 122 nm^2^ in all cases (see the Supplementary Material), which is almost identical to the SASA value obtained before adsorption (124 nm^2^). This shows that the size of the protein does not change after adsorption. We obtain that the protein amino acids in contact with the surface are the same in Simulation 1 (neutral surface) and Simulation 2 (negatively-charged surface and negatively-charged protein), as shown in Figure 1b,c. This result shows that the dominant protein-surface interaction mechanism is the hydrophobic effect, in spite of the protein being negatively charged at this pH. On the contrary, the change of pH from 7 to 4 (Simulation 3, positively-charged protein) increases the number of amino acids in contact with the negatively-charged surface, as seen in Figure 1d. In this case, both hydrophobic and electrostatic interactions favor adsorption, and the protein increases its effective contact with the surface.

Comparing the adsorbed state in Simulations 1 and 2 with the bulk state, we obtain an RMSD of 0.45 nm for both cases (see Figure S2 of the Supplementary Material), which indicates that the relative positions of the protein atoms change due to adsorption. The RMSD between the adsorbed protein onto a negatively-charged surface at pH 7 (Simulation 2) and the final adsorbed structure at pH 4 (Simulation 3) is ∼0.5 nm (see the Supplementary Material (Figure S3)), which indicates a further change in the atomic coordinates due to the pH change. These results for RMSD indicate that the relative atomic positions of the atoms change depending on external conditions, a consequence of the fact that β-casein is a flexible protein. In contrast, the size of the protein remains constant, as indicated by the SASA results (see Figure S4); the available surface area is almost identical in all simulations.

**Figure 2:**
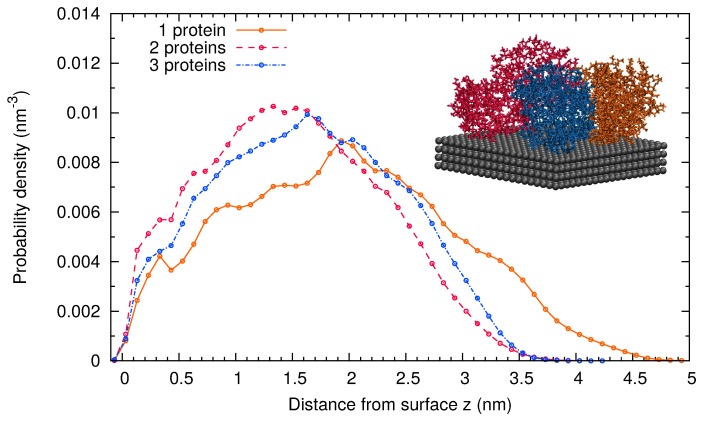
Probability distribution of protein atoms as a function of the distance from the adsorbing surface obtained in MD simulations with one, two or three β-casein adsorbed onto a 45.5 nm^2^ hydrophobic surface and neutral pH. Inset: snapshot of the film formed by three β-casein with each protein shown in a different colors.

Finally, we consider the adsorption of several β-casein proteins at the surface at pH 7 and 298 K (Simulations 4 and 5). This allowed us to explore the role of the protein-protein interaction in the adsorbed layer. A representative snapshot of the final configuration of Simulation 5 with three adsorbed proteins is shown as the inset in Figure 2. It corresponds to an area of 15.18 nm^2^ per protein or equivalently to a mass density of 2.6 mg/m^2^. In the final configuration with three adsorbed proteins, the most hydrophobic region of each protein (see the Methods Section 3) is in contact with the surface or with another protein.

The thickness of the monolayer is about 3 nm, as can be estimated from the atomic probability distribution of the protein backbone (Figure 2). For very small coverages of the surface (simulations with one protein at the surface, equivalent to 0.87 mg/m^2^ mass density), the atomic probability distribution of the protein backbone extends to larger distances. In spite of the significant electrostatic protein-protein repulsion (each protein has a charge of −8e), the protein layer is more compact as the coverage increases. This shows that the overall protein-protein interaction is attractive. The favorable interaction coming from the contact between hydrophobic residues overcomes the electrostatic repulsion.

### 2.2. Comparison of Simulation Results for β-Casein, β-Lactoglobulin and BSA

**Figure 3:**
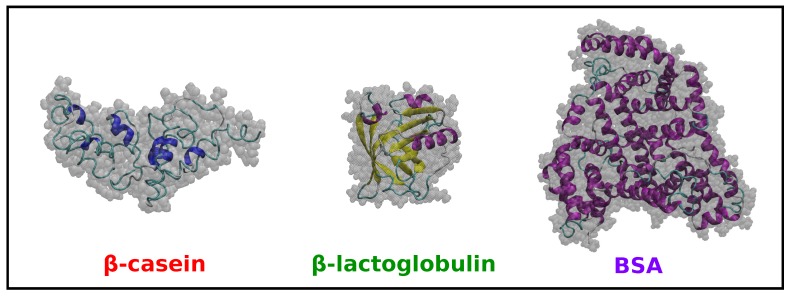
Snapshots of β-casein, β-lactoglobulin and BSA in MD simulations at neutral pH and 298 K (see the text). The structure of the proteins is shown in cartoon representation, and the size of the protein is indicated in grey using a surface representation. These images were made with VMD [29].

In addition to the MD simulations of β-casein discussed in Section 2.1, we have performed simulations for β-lactoglobulin and BSA in implicit solvent at neutral pH and 298 K (see the Methods Section 3 for details). The results are summarized in Table 1, and snapshots of the equilibrated coordinates are shown in Figure 3.

First, we note that the obtained small value for RMSD (Table 1) for β-lactoglobulin and BSA indicates that there is only a very small difference between the equilibrated protein structures (obtained from simulations at 298 K) and the structures from the X-ray crystal structures (available in PDB database). It is also interesting to note that in both cases, the proteins have a larger solvent-accessible surface area (SASA) at 298 K compared with that computed from the X-ray crystal structures. As mentioned before, the X-ray structure of β-casein is not available.

Concerning the size of the proteins (Table 1), we note that BSA has the larger radius of gyration $R_{g}$, whereas β-casein and β-lactoglobulin have similar sizes, as expected from the number of amino acids of each protein (in globular proteins, the radius of gyration scales with the number of amino acids as $R_{g}∼N^{1/3}$) [30].

The shape of the proteins is characterized in Table 1 by the three principal radii of gyration $R_{1}$, $R_{2}$ and $R_{3}$ and the parameters Δ and *S*. β-lactoglobulin is, with a very good approximation, a spherical globular protein since the three principal radii are almost identical, and it has values of Δ and *S* close to zero. In the case of β-casein, one of the principal radii is slightly smaller than the others, but the deviation from a globular shape is small, as indicated by the low values of Δ and *S*. BSA has one of its principal radii larger than the other two (which are very similar), but again, the deviations from spherical shape indicated by Δ and *S* are small.

As in the case of β-casein, we have also employed the resulting coordinates obtained from MD simulations as initial configurations for a PropKa semi-empirical calculation of the isoelectric point and the dependence on the charge of the protein as a function of pH. Although this method can be employed directly from the protein structures available in the databases, we found better agreement with experiments by starting from coordinates corresponding to configurations equilibrated at the temperature of interest (298 K). The obtained isoelectric points are in reasonable agreement with reported experimental values, as shown in Table 1. The full curves of protein charges as a function of pH are given in the Supplementary Material (Figure S1).

We can compare the results obtained for the adsorption of β-casein (Section 2.1) with published simulation results obtained for adsorption of β-lactoglobulin [15] and BSA [27,31]. BSA and β-lactoglobulin spread slightly over the surface when adsorbed, without significant changes in their structure. The obtained RMSD after adsorption (0.3 nm for BSA and 0.15–0.20 nm for β-lactoglobulin) are lower than the RMSD values obtained for β-casein (≈0.5 nm). In the case of BSA, simulations also indicate that the protein does not change significantly even when adsorbing onto small (diameter 6 nm) nanoparticles [27]. These results are consistent with the view of β-casein as the less structured and more flexible of the three proteins considered.

These results correspond to the interaction of a single protein with a surface. In the case of a saturated surface, results for BSA [27] indicate that this protein adsorbs independently over the surface without self-assembling. On the contrary, our results for β-casein films described in the previous subsection indicate a significant interaction between adsorbed proteins, which tend to assemble or aggregate at the surface.

### 2.3. QCM-D and AFM Study of Protein Adsorption onto a Planar Surface

We evaluated the adsorption of the proteins from 1-mg/mL solutions on hydrophobic polystyrene (PS) surfaces. The experiments were performed at pH 6 for BSA and β-lactoglobulin and at pH 7 for β-casein. These pH values were chosen near the pI of the proteins (see Table 1) to maximize the adsorption by decreasing the intermolecular electrostatic repulsion among proteins, but above this value to avoid protein aggregation in solution. In this way, the protein-protein electrostatic repulsion is minimized, and the formation of a well-packaged protein layer is possible, as seen in our MD simulations reported in Section 2.1. At the employed pH, the PS surface is negatively charged due to adsorption of OH^−^ with an estimated charge of −0.039 e/nm^2^ [32].

**Figure 4:**
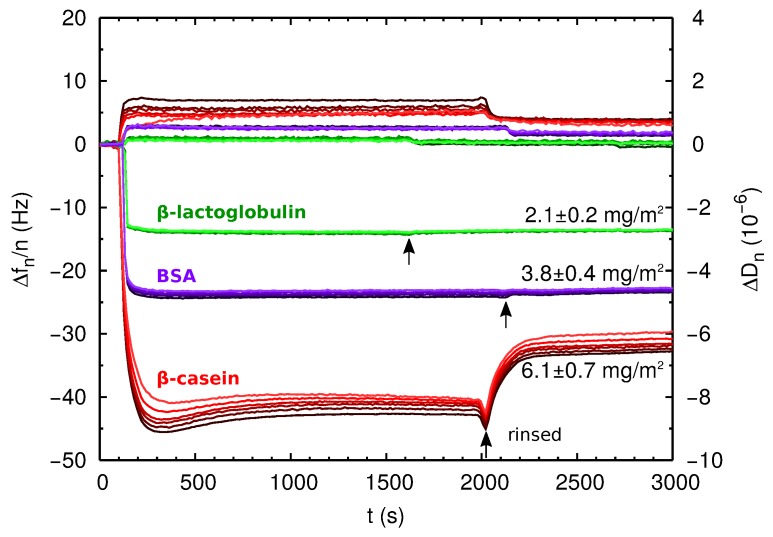
QCM-D measurements for direct adsorption experiments of proteins onto negatively-charged PS hydrophobic surfaces. The curves labeled with the protein names correspond to the changes in resonance frequency $\Delta f_{n}/n$ of the QCM crystal as a function of time due to the addition of 1 mg/mL protein solution. We also show the energy dissipation $\Delta D_{n}$ measured during the same experiments (unlabeled curves). Black arrows indicate the time at which the crystal is rinsed with protein-free buffer solution. The overtones from n=3 to n=13 are represented in different tonalities from dark to pale. The adsorbed mass indicated in the figure is obtained using Equation (6) as explained in the main text.

In Figure 4, we show the QCM-D raw data obtained for the three proteins. Changes in resonance frequency ($\Delta f_{n}$) and dissipation ($\Delta D_{n}$) were observed upon protein injection in all cases, indicating protein adsorption. Clear qualitative differences can be established between the adsorption of BSA and β-lactoglobulin on one side and β-casein on the other side. For BSA and β-lactoglobulin, $\Delta f_{n}/n$ and $\Delta D_{n}$ reach their steady-state values within a few minutes after protein injection. These values did not change when the films were rinsed with protein-free solution (see Figure 4). The values of $\Delta f_{n}$ show no significant dispersion with *n* for the overtones from n=3 to n=13, and the low dissipation condition is verified. These results indicate that for BSA and β-lactoglobulin, we obtain a rigid film, and the Sauerbrey equation, Equation (6), can be safely employed. The obtained areal mass density $m_{f}$ is given in Table 2, and it is also indicated in Figure 4. In this situation of large coverage of the surface, we estimate the area per adsorbed protein as:

(1) $$a_{p}=M_{W}/m_{f},$$

where $M_{w}$ is the molecular weight of the protein. The results are given in Table 2. It is interesting to compare the obtained values of $a_{p}$ and the dimensions of the proteins obtained in simulations (Table 1). In the case of β-lactoglobulin, which is essentially spherical, its cross-sectional area can be estimated as $a_{c}\approx \pi R_{g}^{2}\approx 14$ nm^2^. This is very close to the value of $a_{p}\approx 14.4$ nm^2^, which indicates that the proteins form a compact layer almost without interstices between the proteins. In the case of BSA (which has a more ellipsoidal shape), the cross-sectional area in upright orientation can be estimated as $a_{c}\approx \pi \left(R_{2}^{2}+R_{3}^{2}\right)$ nm^2^≈27 nm^2^. This is also very close to the estimated area per molecule of $a_{p}\approx 29$ nm^2^ reported in Table 2, indicating again the formation of a compact monolayer of protein.

On the contrary, the case of β-casein has several distinctive features. At short times after protein injection, an overshoot in $\Delta f_{n}$ and $\Delta D_{n}$ was observed before reaching the equilibrium value. This overshoot can be due to protein reorganization at the adsorbed film. Furthermore, when the film is rinsed with protein-free solution, there is another overshoot followed by a drop of $\Delta f_{n}/n$ and $\Delta D_{n}$. This behavior can be due to the formation of a multilayer film. Thus, the first overshoot, may correspond to protein reorganization due to the build-up of the multilayer, and the second overshoot (observed when the film is rinsed with free-protein solution) may correspond to removal of weakly-adsorbed proteins on the top layers of the multilayer. There is also a measurable overtone dispersion of the values of $\Delta f_{n}$ with *n*, an effect that indicates that the film is not purely elastic [33]. In spite of the dispersion in $\Delta f_{n}/n$, we can obtain an estimation of the film areal mass density using Equation (6), but in this case, the calculation is less accurate and has to be considered with caution. The result is given in Table 2, and it is also indicated in Figure 4. Another reason for the large effective thickness of the protein film could be an important amount of water associated. This possibility can be further analyzed by measuring the height of the film layer in dry conditions.

**Figure 5:**
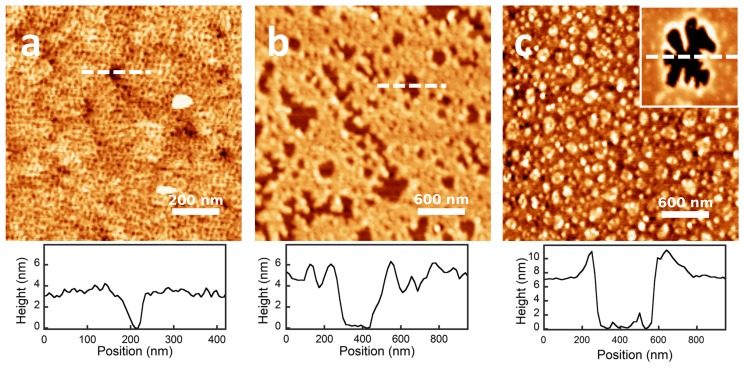
Height AFM micrographs in air of the dry protein films adsorbed onto SAM gold surfaces of CH_3_. (**a**) β-lactoglobulin; (**b**) BSA and (**c**) β-casein films. A typical height profile for each sample is presented. Values for the thickness of the films are compiled in Table 2. Scales are indicated in the figure.

The thickness of the dry protein films was measured in air by AFM. The AFM micrographs (Figure 5) show that the proteins are homogeneously distributed over the surface. The results for the film thickness can be observed in the height profiles of the figure where the thickness is measured from the free-protein planar surface (0 nm) until the stable value reached by the protein film. The values are also presented in Table 2. In the case of β-lactoglobulin and BSA, the measured height is consistent with a film made of a protein monolayer. On the contrary, for the case of β-casein, the height of the dry protein film measured by AFM (≈7 nm) is more than two-times the value obtained in our MD simulations of a protein monolayer (≈3 nm) (see Figure 2). Therefore, AFM measurements support the idea that the β-casein film contains at least two protein layers.

**Figure 6:**
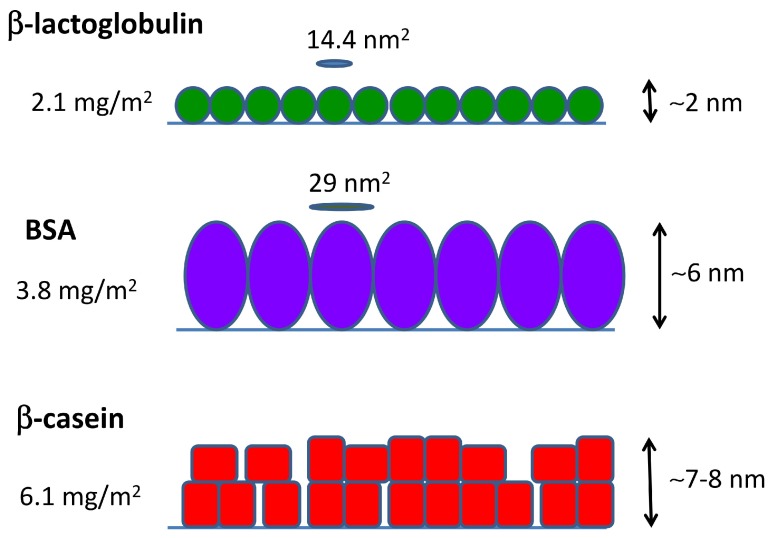
Cartoon indicating the possible arrangement of the different proteins in the protein films, summarizing our results of QCM-D and AFM measurements. The interpretation on which the cartoon is based also takes into account the protein dimensions and shape obtained from simulations (see the text for details).

Summing up all of the evidence coming from MD simulations and QCM-D and AFM measurements, we can obtain a full picture of the adsorption of the three studied proteins, as given in Figure 6. β-lactoglobulin forms a monolayer that can be represented by a compact arrangement of spherical objects. BSA also adsorbs as a compact monolayer, but formed by slightly elongated objects in upright orientation. β-casein films consist of a multilayer of deformable objects that tend to be in close contact.

Overall, the arrangements proposed in Figure 6 in the case of BSA and β-lactoglobulin are consistent with previously-reported experimental results (see [13,34,35] for BSA and [13,36,37] for β-lactoglobulin). In the case of β-casein films, we can found both examples of monolayer or multilayer formation in the literature [34,36,38]. Earlier β-casein QCM-D studies reported areal masses in agreement with our results [39,40]. Experimental works based on the batch depletion method [41,42] or ellipsometry [43,44] reported values of 2–3 mg/m^2^ for a full-coverage monolayer of β-casein. These results also support our interpretation that the value of 6.1 mg/m^2^ obtained by QCM corresponds to multilayer formation.

**Figure 7:**
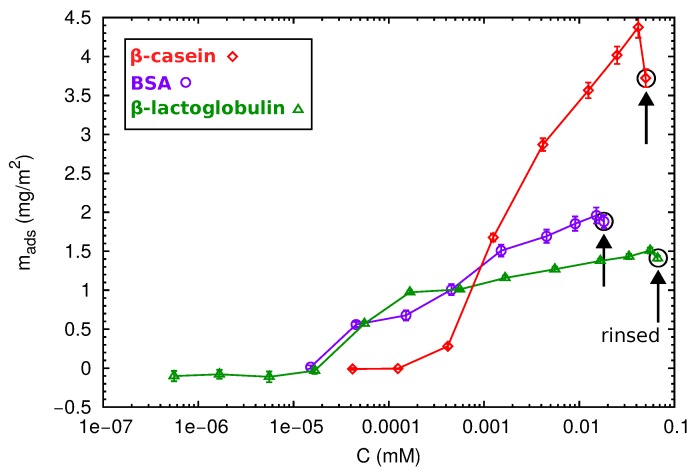
Adsorption isotherms obtained from QCM-D measurements for protein adsorption onto PS hydrophobic surfaces and pH 6 (β-lactoglobulin and BSA) or pH 7 (β-casein). The last data point was obtained after rinsing with protein-free buffer solution, as indicated in the figure.

After the direct adsorption experiments, we have also considered adsorption at incremental concentration isotherms for the different proteins. Increasing protein concentrations were progressively injected into the QCM-D cell in contact with the PS surface, and the adsorbed areal mass as a function of the protein concentration was calculated by the Sauerbrey equation (Equation (6)). The obtained results are shown in Figure 7.

At first sight, different behaviors can be observed. The adsorption of the globular proteins BSA and β-lactoglobulin starts at 5 × $10^{-5}$ mM, reaching a maximum value of 2 and 1.5 mg/m^2^, respectively. After rinsing with protein-free buffer solutions, there was no desorption indicating that the proteins are well attached to the surface. In contrast, the β-casein needs a minimum concentration of 5 × $10^{-4}$ mM to produce a significant deposition onto the surface. In this case, the amount of adsorbed protein progressively increases with protein concentration, and no saturation is observed. This is in agreement with the multilayer formation. After one rinse cycle, loosely-adsorbed protein molecules leave the surface, and the adsorbed mass decreases.

The highest protein concentration injected during the isotherm experiments corresponds with the concentration used in the direct adsorption discussed before (1 mg/mL). However, the total amount of protein in a direct adsorption (numbers shown in Figure 6) is higher than that obtained in the isotherm adsorption (Figure 7). This phenomenon has been observed for many proteins, and it is usually interpreted as follows [12]. At a high concentration, proteins are cooperatively adsorbed, and the packaging with neighboring proteins is improved. At low concentrations, individually-deposited proteins cover a higher area than collectively-adsorbed proteins. In absence of re-structuring mechanisms at the surface, there is less available space for the further addition of proteins, and the final coverage will be lower. This effect also suggests that protein adsorption is an out of equilibrium process, so the concept of the adsorption isotherm has to be considered with care [45].

### 2.4. Electrokinetics of Adsorbed Protein Layers

**Figure 8:**
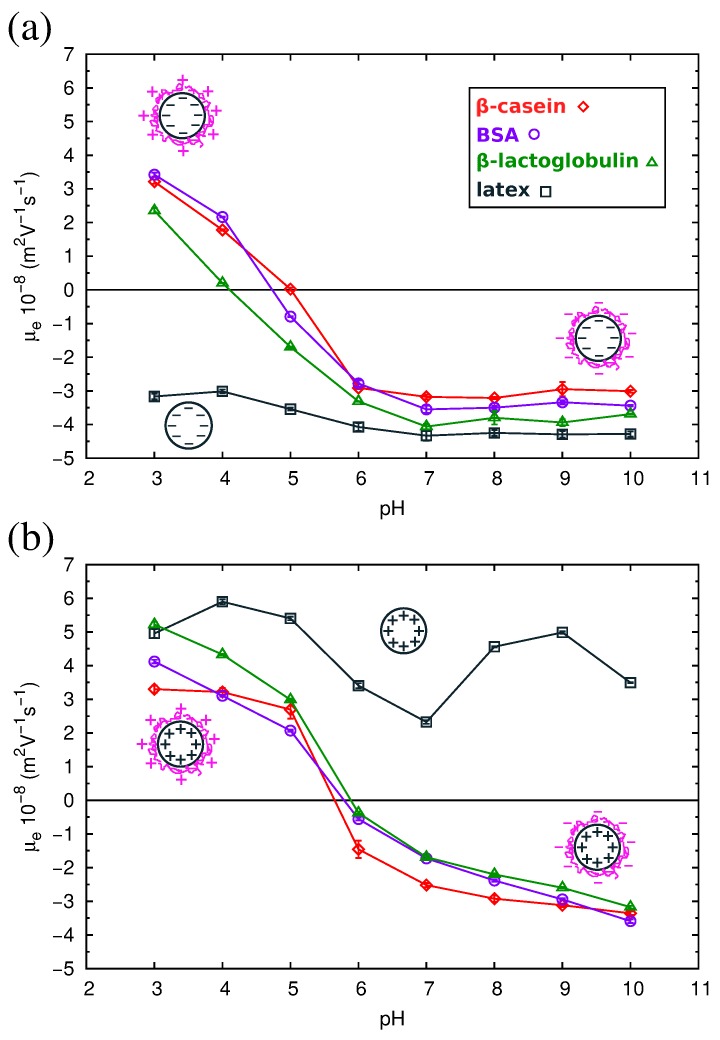
Electrophoretic mobility ($\mu_{e}$) measurements as a function of pH of the protein-coated latex complexes formed with the three proteins and (**a**) anionic latex and (**b**) cationic latex. We also show the mobility for the bare latexes without adsorbed protein for a reference. The solid lines are guides to the eye.

QCM results have allowed us to understand the structure of the adsorbed protein films. However, from this technique, we cannot obtain information about the electrical state of the adsorbed proteins, an important parameter that influences the final properties of the protein films. For this reason, we studied complexes formed by the physical adsorption of the three proteins over two different kinds of polystyrene (PS) latex microspheres (anionic and cationic). The conditions of the adsorption process were identical to those corresponding to the direct adsorption experiments over flat PS surfaces reported in Section 2.3. For the characterization of the electrical state of the complexes, we measured their electrophoretic mobility ($\mu_{e}$) as a function of pH. The results are shown in Figure 8, and the isoelectric points of the complexes are summarized in Table 3.

As can be observed in the figure, the $\mu_{e}$ of the complexes presents positive values at acid pHs and negative values at alkaline pHs. For the three proteins, the isoelectric points of the complexes obtained with both anionic or cationic latexes differ by about 1 pH unit from the pI of the proteins in solution (Table 3). The pI of the complexes is displaced to more acid values in the case of the anionic latex and more alkaline values for the cationic latex. Previous electrokinetic studies [46,47,48] also reported that at large amounts of adsorbed proteins, the pI values of the complexes are close to the pI of the protein molecules.

As can be seen in Figure 8, the absolute values of $\mu_{e}$ of the protein-coated PS particles are smaller than for the latex particles, implying a reduction of the electrokinetic charge after protein adsorption. This is found even in the cases in which both the latex and the protein have charges of the same sign. For example, in Figure 8a, we see that for the anionic latex at alkaline pHs (which correspond to negative charges of the proteins; see Table 1), the absolute value of $\mu_{e}$ follows the order: bare latex > β-lactoglobulin > BSA > β-casein. These counter-intuitive results have been also reported in previous works [49,50]. The causes of this extensively-observed behavior are still unclear [24,51,52]. On the one hand, it has been suggested that the latex surface is not fully charged for a high coverage of protein, since the charge of an ionizable group is strongly dependent on the dielectric constant [53,54,55], and the latex is in contact with a protein film of low dielectric constant. On the contrary, in water, the latex charge is maximized, being in contact with a solvent with a high dielectric constant. Thus, the effective contribution of the latex to the charge of the complex (and the electrokinetic motion) is reduced upon protein adsorption. On the other hand, we have to take into account the possibility of the adsorption of ions from the buffers over strongly-charged surfaces. This ion condensation process could be partially responsible for the observed charge reduction of the complexes.

**Figure 9:**
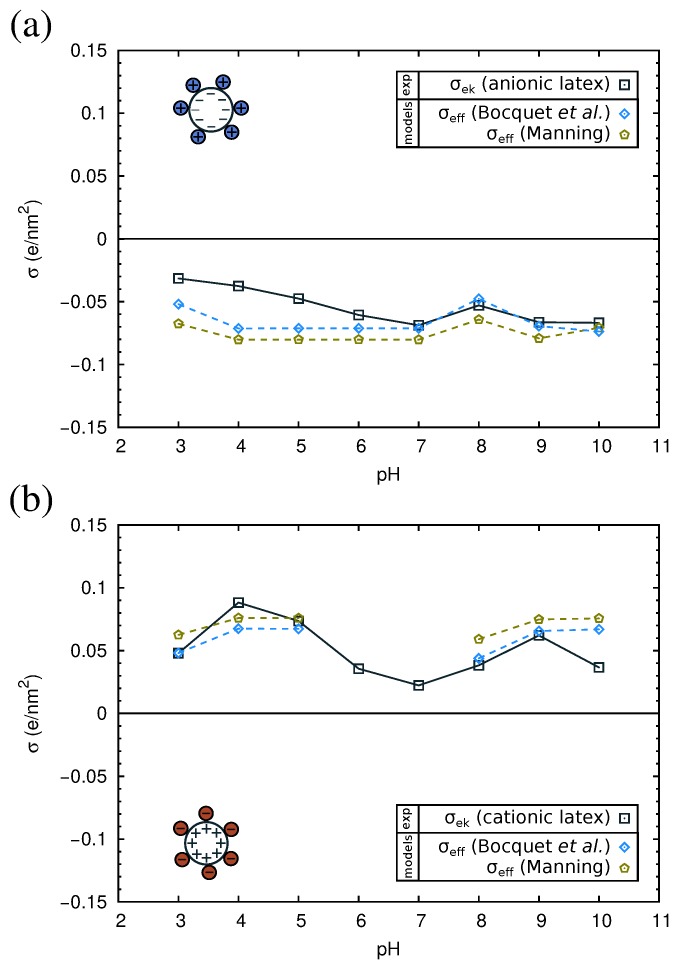
Electrokinetic charge ($\sigma_{ek}$), obtained by Ohshima’s equation (Equation (5)), and effective charge ($\sigma_{eff}$) taking into account ion condensation (the Manning model and the Bocquet et al. model) for (**a**) anionic and (**b**) cationic latex. The solid and dashed lines are used for guiding the eye.

In order to interpret the obtained experimental results, we will now analyze them using current theories of ion condensation. Let us first consider the bare latex particles. The surface charge density ($\sigma_{0}$) measured by direct titration is −0.6 e/nm^2^ for the anionic and +1.1 e/nm^2^ for the cationic latex. The electrokinetic charge density ($\sigma_{ek}$) of the latexes as a function of the pH is shown in Figure 9, as computed from the measured electrophoretic mobility using Ohshima’s equation (Equation (5)). As can be observed, for both the anionic and the cationic latex, the electrokinetic charge $\sigma_{ek}$ is one order of magnitude smaller than $\sigma_{0}$.

As mentioned before, the electrokinetic charge comes from the charge of the colloids and the contribution due to the adsorbed counterions. Our results suggest that there is a substantial reduction of their electrokinetic charge due to ion condensation [56,57,58]. We denote this reduced charge as the effective charge ($\sigma_{eff}$). In order to calculate $\sigma_{eff}$, we will consider two different theories of ion condensation, the electrostatic theory proposed by Manning [59] and the ion-correlation theory proposed by Bocquet et al. [60]. According to these theories, if the bare charge of the colloid ($\sigma_{0}$) surpasses a critical charge density $\sigma_{crit}$, there is a strong condensation of counterions, and the effective charge is given by $\sigma_{eff}=\sigma_{crit}$. If $\sigma_{0}$ > $\sigma_{crit}$, the effective charge density is independent of the bare charge of the colloid, and it is only dependent on the ionic medium. The explicit equations and procedures for the calculation of $\sigma_{crit}$ differ in each theory. In the Manning theory, $\sigma_{crit}$ is given by:

(2) $$\sigma_{crit}=-\frac{e\left(1+\kappa a\right)\;\ln\left(\kappa \lambda_{B}\right)}{2\pi |z|\lambda_{B}a},$$

and in the theory proposed by Bocquet et al. [60], it is given by:

(3) $$\sigma_{crit}=\frac{e(1+\kappa a)}{\pi a\lambda_{B}}.$$

In Equations (2) and (3), *e* is the elementary charge, *a* is the radius of the colloid, *z* the valence of the counterions, κ is the Debye–Hückel constant and $\lambda_{B}=e^{2}/4\pi \varepsilon_{0}\varepsilon_{r}k_{B}T$ (≈0.71 nm) is the Bjerrum length ($\varepsilon_{0}$ is the vacuum permittivity, $\varepsilon_{r}$ the dielectric constant of water, $k_{B}$ Boltzmann’s constant and *T* the temperature).

These equations are valid for colloids immersed in solution of symmetrical electrolytes, which is the case of the buffered solutions used in this study, except for the case of the cationic latex at pH 6 and 7 (see the Supplementary Material).

The results of $\sigma_{crit}$ are given in Table S3 in the Supplementary Material. In all cases, $\sigma_{0}$ is bigger than $\sigma_{crit}$. Therefore, $\sigma_{eff}=\sigma_{crit}$. In Figure 9, we compare the experimental values of $\sigma_{ek}$ with the predictions of $\sigma_{eff}$. As can be observed in the figure, the results of both theories are close to our experimental values. Thus, ion condensation appears to be a good hypothesis to explain the reduction of the apparent surface charge density in the case of bare latex particles.

The effective charge of the latex is only a small fraction (∼5–10%) of its bare charge. With this result in mind, we can discuss now the results for the latex-protein complexes.

**Figure 10:**
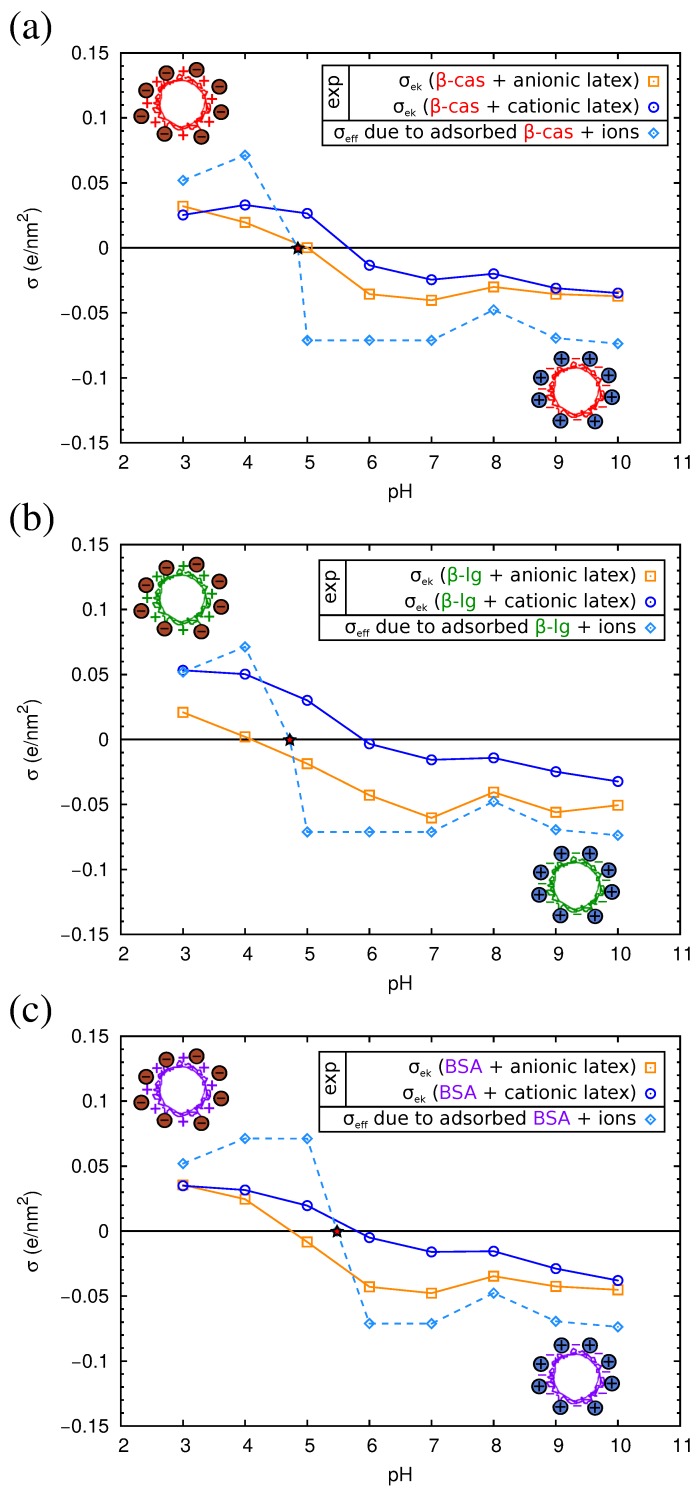
Electrokinetic charge ($\sigma_{ek}$), obtained by Ohshima’s equation (Equation (5)), for the complexes consisting of proteins + anionic or cationic latex and effective charge ($\sigma_{eff}$) due to adsorbed protein taking into account ion condensation (Bocquet et al. model) for (**a**) β-casein; (**b**) β-lactoglobulin and (**c**) BSA. The star indicates the theoretical pI of the proteins from Propka calculations. The solid and dashed lines are used for guiding the eye.

In Figure 10, we show the electrokinetic charge density $\sigma_{ek}$ of the complexes computed from the experimental electrophoretic mobility $\mu_{e}$ using Equation (5). For each one of the three considered proteins, we have two experimental $\sigma_{ek}$ curves, corresponding to complexes made with the anionic or the cationic latex. In all cases, these two curves give very similar values of $\sigma_{ek}$, except near the isoelectric point of the complexes. This fact suggests that the adsorbed protein film is the dominant component in the electrokinetic charge of the complex and reinforces the hypothesis that the electrostatic charge of the latex is hardly manifested due to the low permittivity of the protein medium. Thus, in this case, we can assume that $\sigma_{0}$ of the latex-protein complex is given by $\sigma_{0}=Q_{p}/a_{p}$, where $Q_{p}$ is the protein charge (which depends on the pH), and $a_{p}$ is the area per adsorbed protein. The PropKa calculations discussed in Section 2.2 give us a theoretical prediction of the charge for each protein, $Q_{p}$ as a function of pH (see Figure S1 in the Supplementary Material). From the QCM-D experiments, we can have a good estimation of the area per adsorbed protein, $a_{p}$ (Table 2). With these data, we can evaluate now the role of ion condensation in the electrokinetic behavior of the complexes. We will employ only the theory of Bocquet et al., Equation (3), which seems to be in better agreement with our experimental values. First, we verified for each pH that $\sigma_{0}$>$\sigma_{crit}$. Thus, the effective charge for the protein film is given by $\sigma_{eff}=\sigma_{crit}$. This effective charge accounts for the adsorbed protein film and the condensed counterions from the buffer.

The dependence on $\sigma_{eff}$ and $\sigma_{ek}$ with pH is presented in Figure 10. As can be seen in the figure, the absolute value of $\sigma_{eff}$ is of the order of 0.05 e/nm^2^ at both acidic or basic pH. The theoretical values of $\sigma_{eff}$ that appear in Figure 10 correspond to the complexes of the anionic latex. Almost identical results were obtained for the cationic latex (not shown). According to Equation (3), the only difference in $\sigma_{eff}$ is determined by the radius of the colloid.

As seen in Figure 10, at each pH (except near the pI) and for all complexes, the absolute value of $\sigma_{eff}$ is larger than $\sigma_{ek}$. This result suggests that in addition to the ion condensation predicted by the model of Bocquet et al., there may be other effects that further reduce the charge of the complex. Several causes can be evoked. First, the charge of the proteins in bulk solution, as computed in the modeling Section 2.2, may differ from the charge of adsorbed proteins. Ionizable amino acids in contact with the low dielectric surface may have lower charge than expected in water (which has a high dielectric constant). Another important factor could be the heterogeneous nature of the interface, which is neglected in the model. Computer simulations show that the binding affinity of ions is higher in model interfaces containing charged chemical groups as compared with idealized homogeneously-charged surfaces with the same charge density [61].

## 3. Materials and Methods

### 3.1. Experimental

#### 3.1.1. Reagents

All of the products were of analytical grade and used as received. The salts were purchased from Scharlau and proteins from Sigma Aldrich: bovine serum albumin (BSA) (purity ≥ 98%), β-lactoglobulin (purity ≥ 90%, mixture of A and B genetic variants) and β-casein (purity ≥ 98%). According to the supplier, the molar mass of each protein is 66 kDa (BSA), 18.4 kDa (β-lactoglobulin) and 23.6 kDa (β-casein), and their pI are 5.3 (BSA), 5.1 (β-lactoglobulin) and 4.6–5.1 (β-casein). Water used in all experiments was double distilled and deionized (DDI) with a Milli-Q Water Purification System (Millipore).

Several buffers of low ionic strength (lower than 2 mM) were used (see Table S1): pH 4 and 5 were buffered with acetic acid (AcH), pH 6 and 7 with monosodium phosphate (NaH_2_PO_4_) and pH 8, 9 and 10 with boric acid (H_3_BO_3_). In each case, the pH was adjusted by adding a few droplets of a NaOH solution. In addition, we used a buffered solution at pH 7 with Bis-Tris in order to dissolve the β-casein [62]. The pH of this solution was adjusted by adding dilute HCl; the ionic strength of the solution was of 2.4 mM. Non-buffered solution at pH 3 was prepared by adding dilute HCl to DDI water. The composition of the buffered solutions is indicated in the Supplementary Material.

#### 3.1.2. Polystyrene Latex Microspheres

Negative and positive polystyrene (PS) microspheres were used. The anionic latex (surface charge density −9.6 μC/cm^2^ (−0.6 e/nm^2^); mean diameter 138 ± 7 nm with high monodispersity) was synthesized in the laboratory of Granada; sulfonate groups provide its anionic nature. The synthesis process and characterization can be found in [63,64] The positive polystyrene latex was supplied by IKERLAT Polymers (surface charge density 17.4 μC/cm^2^ (1.1 e/nm^2^); mean diameter 475 ± 4 nm). Its positive charge is due to amine groups. The diameter of the microparticles was determined by TEM, while the surface charge density was obtained by direct titration.

#### 3.1.3. Protein Adsorption onto Latex Microspheres

Protein-coated PS microspheres were prepared by physical adsorption. First, the desired protein was dissolved in a buffered solution to get a concentration around 1 mg/mL. Both BSA and β-lactoglobulin were prepared in pH 6 buffer (monosodium phosphate); however, β-casein is not fully dissolved in these conditions. For this reason, β-casein was dissolved in pH 7 buffer (Bis-Tris), where the protein presents the monomeric form [62]. The β-casein concentration employed was always lower than the critical micelle concentration (CMC) [65,66]. The solutions were stirred during 1 h to ensure complete solubilization of the proteins. The concentration of the protein in the solution was determined by UV absorption according to the Beer–Lambert law. The absorbance of our samples was measured at 280 nm in a Spectronic Genesys 5 device. The extinction coefficients of the proteins are: ε(BSA) = 0.66 mL/mg·cm, ε(β-lactoglobulin) = 0.96 mL/mg·cm and ε(β-casein) = 0.46 mL/mg·cm [24,51,52].

The latex microparticles were mixed with the protein solution (0.3 mg/mL) at the ratio of 8 mg of protein per m^2^ of latex surface. The incubation was performed in a shaking water bath at 25 °C during 21 h, as performed in an earlier study [67]. Then, the solution with the complex (latex-protein) was centrifuged at 14,000 rpm and 20 °C during 20 min (Hettich Mikro 220R) to remove the non-adsorbed proteins. The supernatant was discarded, and the pellet was redispersed in the same pH buffer of the adsorption process. No desorption was found during one week after the incubation. Latex-protein complexes were discarded and no longer used after that period.

#### 3.1.4. Electrokinetics

The measurements of size and electrophoretic mobility were carried out using a Zetasizer Nano Z device (Malvern Instruments). The electrophoretic mobility ($\mu_{e}$) was measured for the PS latexes and the complexes (latex-protein). In every measurement, the microparticles were diluted in the desired solution using a concentration of $10^{10}$ particles/cm^3^. The data obtained were the average of three measurements, and the standard deviation was always lower than 5%.

From the measured electrophoretic mobility ($\mu_{e}$), we have computed the ζ-potential and the electrokinetic charge density ($\sigma_{ek}$), which is the electric charge responsible for the electrophoretic motion. We have employed the following accurate analytical approximations valid for spherical colloids [68,69]:

(4) $$\begin{array}{c} \mu_{e}=\frac{2\varepsilon_{0}\varepsilon_{r}\zeta }{3\eta }\left(1+\frac{1}{2{\left[1+2.5/\left\{\kappa a\left(1+2e^{-\kappa a}\right)\right\}\right]}^{3}}\right)\\ -\frac{2\varepsilon_{0}\varepsilon_{r}\zeta }{3\eta }{\left(\frac{ze\zeta }{k_{B}T}\right)}^{2}\left[\frac{\kappa a\{\kappa a+1.3\exp(-0.18\kappa a)+2.5\}}{2{\left\{\kappa a+1.2\exp\left(-7.4\kappa a\right)+4.8\right\}}^{3}}\right.\\ \left.+\left(\frac{m_{+}+m_{-}}{2}\right)\frac{9\kappa a\{\kappa a+5.2\exp(-3.9\kappa a)+5.6\}}{8{\left\{\kappa a-1.55\exp\left(-0.32\kappa a\right)+6.02\right\}}^{3}}\right] \end{array}$$

(5) $$\begin{array}{c} \sigma_{ek}=\frac{2\varepsilon_{0}\varepsilon_{r}\kappa k_{B}T}{ze}\sinh\left(\frac{ze\zeta }{2k_{B}T}\right)\left[1+\frac{1}{\kappa a}\frac{2}{\cosh^{2}\left(ze\zeta /4k_{B}T\right)}\right.\\ \left.+\frac{1}{{\left(\kappa a\right)}^{2}}\frac{8\ln[\cosh\left(ze\zeta /4k_{B}T\right)]}{\sinh^{2}\left(ze\zeta /2k_{B}T\right)}\right] \end{array}$$

In Equations (4) and (5), η is the viscosity of water, $\varepsilon_{0}$ is the vacuum permittivity, $\varepsilon_{r}$ is the dielectric constant of water, *e* is the elementary charge, *z* is the valence of the symmetrical electrolyte, $k_{B}$ is Boltzmann’s constant, *T* is the absolute temperature, κ is the inverse of the Debye–Hückel length, *a* is the radius of the colloid and $m_{+}$ and $m_{-}$ are dimensionless ionic drag coefficients (see [69]).

These expressions are valid for symmetrical electrolytes, which was the case for all of the buffers used in this study, except for pH 6 and 7. We have calculated the Debye–Hückel length using monovalent and multivalent ions (when present), but in Equations (4) and (5), we have always considered *z* = 1. This assumption is reasonable, since the concentration of monovalent ions is always much larger than that of multivalent ions (if present at all) in the buffers used in this study.

#### 3.1.5. Quartz Crystal Microbalance

The quartz crystal microbalance with the dissipation monitoring (QCM-D) device (Q-sense E1 by Biolin Scientific) is a very sensitive tool, which can be employed to determine the adsorbed mass in protein films [70]. It is based on a piezoelectric system in which an alternating voltage is applied over a quartz crystal, producing mechanical vibrations close to its resonant frequencies. In QCM-D, the applied voltage is intermittently switched on and off, and the oscillations are left to decay freely. The voltage generated during the mechanical oscillations in the piezoelectric is recorded and spectrally analyzed, yielding two parameters per odd overtone *n*, the resonance frequency $f_{n}$ and the dissipation $D_{n}$ defined from the bandwidth $\Gamma_{n}$ as $2\Gamma_{n}=D_{n}f_{n}$. The dissipation $D_{n}$ is also the inverse of the quality factor of the resonance peak $D_{n}=Q_{n}^{-1}$. Any small mass deposited over the crystal results in a change in the resonance frequencies $\Delta f_{n}=f_{n}-f_{0,n}$ of the coated crystal. The change in resonance frequencies $\Delta f_{n}$ can be translated into the areal mass density if the variation in $\Delta f_{n}/n$ as a function of *n* (the overtone dispersion) and the dissipation value $\Delta D_{n}$ are both low [70]. In this case, the adsorbed mass of the film $m_{f}$ is proportional to the change in the resonance frequencies $\Delta f_{n}/n$, and it can be calculated by using the Sauerbrey equation [71]:

(6) $$m_{f}=-C\frac{\Delta f_{n}}{n},$$

where *C* is the mass sensitivity constant, which depends on the material properties of the quartz crystal and on its fundamental resonance frequency. In our case, *C* = 18 ng ${\mathrm{cm}}^{-2}$ ${\mathrm{Hz}}^{-1}$, with a fundamental frequency of $f_{F}$ = 5 MHz.

It is important to recall here that the film mass $m_{f}$ calculated from Equation (6) is the wet mass, that is it includes the solvent associated with the adsorbed film. The contribution of the surrounding liquid to the frequency response is non-linear, and it decreases substantially with increasing coverage.

Our experiments were performed using crystals with gold electrodes that were hydrophobically modified with the following protocol. First, the crystals were cleaned with absolute ethanol (99.8% of purity) and gently dried with N_2_ gas. Afterwards, they were irradiated with ultraviolet light in an UV/ozone cleaner for 30 min and rinsed again with ethanol and dried with N_2_ gas. After the cleaning process, the gold surfaces were coated with CH_3_ terminated self-assembled monolayers, by 4-h exposure to a solution of 1 mM of 1-octadecanethiol (98% of purity, Sigma Aldrich) in absolute ethanol. Finally, the surfaces were cleaned with ethanol to remove non-adsorbed material and dried with N_2_ gas. The thiol groups form a chemical bond with the gold surface. After that, the modified gold crystal were coated with polystyrene (250 kg/mol, ACROS Organics) solution in toluene 5%*w*/*w* by spin-coating (at 3500 rpm during 60 s) [32]. This technique enables obtaining uniform thin polymer films. After PS coating, the substrates were annealed at 95 °C for 12 h to remove the residual solvent and release any mechanical stress built up during the spin-coating process. Under these conditions, a 300 nm-thick PS layer is obtained. The hydrophobicity of the surface was evidenced by a water contact angle of 90° and low hysteresis. The PS surface does not have charged chemical groups, but at neutral pH, it is negatively charged due to the adsorption of OH^−^. The estimated charge for this surface is −0.039 e/nm^2^ [32]. After each experience, the surface was rinsed with toluene removing the PS film and recovering the gold-CH_3_ surface. Then, the crystal is ready to be reused [72].

Once the quartz crystal was assembled into the cell of the QCM, the substrate was exposed to the desired buffer solution until a stable signal was reached. At this point, we take the reference resonance frequencies and dissipations ($\Delta f_{n}/n$ = 0 and $\Delta D_{n}$ = 0). Data of adsorbed mass are obtained in one experiment as the mean value of six measurements for the different harmonics of the resonance frequency. After that, we performed two types of experiments: direct adsorption experiments and isotherm determination. In the direct adsorption experiments, a 1-mg/mL protein solution is injected at 25 °C. After 30 min of adsorption, the cell was rinsed with the protein-free buffer solution in order to remove the non-adsorbed proteins. In the adsorption isotherm experiments, the measurements were obtained by injecting solutions with increasing protein concentrations. The largest concentration considered was 1 mg/mL. Again, after adsorption at the largest concentration, the cell was rinsed with the protein-free buffer solution in order to remove the non-adsorbed proteins. All of these experiments were performed at pH 6 buffer (monosodium phosphate) for BSA and β-lactoglobulin or at pH 7 buffer (Bis-Tris) for the β-casein.

#### 3.1.6. Atomic Force Microscopy

The topography of the protein films were investigated in air by AFM (Icon, Bruker) using Si tips on rectangular Si cantilevers (RTESP, Bruker). The proteins were adsorbed onto hydrophobic surfaces at 1 mg/mL in buffered pH conditions as previously explained (pH 6 for BSA and β-lactoglobulin, and pH 7 for β-casein) and room temperature. Ultra-flat gold surfaces were obtained by gold deposition onto mica [73]. After gluing the gold-coated mica on silicon wafers (gold-side down), the mica was removed with tetrahydrofuran (Sigma Aldrich). The gold substrate obtained was rinsed generously with absolute ethanol (99.8% of purity) and gently dried with N_2_ gas. Immediately after, the gold surface was coated with CH_3_ terminated self-assembled monolayers (SAM), by 4-h exposure to a solution of 1 mM of 1-octadecanethiol (Sigma Aldrich) in absolute ethanol. Finally, the substrates were rinsed again with absolute ethanol and dried with N_2_ gas. We obtained a hydrophobic surface for the protein adsorption at the same conditions that in the QCM experiments (the amount of adsorbed protein over the PS surface was identical that over the gold-CH_3_ surface).

### 3.2. MD Simulations

#### 3.2.1. Models and Force Fields

In all our simulations, the proteins were modeled with all-atomic detail. The atomic coordinates of BSA and β-lactoglobulin were obtained from the crystallographic structures deposited in the Protein Data Bank [74] with the PDB codes 4OR0 and 3NQ9, respectively [18,26]. The crystallographic structure of the β-casein is unknown because it is a disordered protein. In this case, we have obtained the atomic coordinates from a 3D model available in the Database of Comparative Protein Structure Models “ModBase” Code 2iw3A. The atomic coordinates available in these database are developed by an automated computational method that performs fold assignments and alignments based on theoretical models [28]. In these protein structures, all missing hydrogen atoms were added using the VMD software [29] with the amino acid protonation states corresponding to neutral pH. After adding the missing hydrogen atoms, our structures had 9207 atoms for BSA, 2584 atoms for β-lactoglobulin and 3124 atoms for β-casein. The charge of these protein structures at pH 7 are −16e for BSA, −8e for β-lactoglobulin and −8e for β-casein.

The force field employed for the protein simulations was CHARMM27 [75,76], modified to account for water as an implicit solvent, according to the generalized Born implicit solvent (GBIS) model [77]. This model has been successfully used in MD simulations of proteins, reducing the cost of expensive explicit solvent simulations. According to the GBIS model, the electrostatic interaction between atoms is described by the Poisson–Boltzmann equation taking into account the Born radius of each atom. This parameter quantifies, for each atom, its exposure to solvent and, therefore, its dielectric screening from other atoms. The model also includes a nonpolar (hydrophobic) energy contribution from implicit solvent. This contribution is proportional to the solvent-accessible surface area calculated using the linear combination of pairwise overlap (LCPO) method [78]. It is important to recall here that, since we are using an implicit solvent model, the nominal simulation times that we will be reporting in the simulations cannot be compared with real experimental times or with simulation times of simulations with explicit water. The motion of the protein atoms in implicit water is much faster than the motion of the protein atoms in explicit water. Depending on the particular case, atomic motion in implicit models, such as the GBIS employed here, are between 2- and 20-times faster than the more realistic times estimated by simulations with explicit water [79]. Therefore, the nominal simulation times shown in the time evolution of the magnitudes reported in the Supporting information must be multiplied by a factor between 2 and 20 to be compared with real times with explicit water.

In the case of the β-casein, we also studied its adsorption onto a hydrophobic planar substrate (both neutral and negatively charged) by MD simulations. The hydrophobic substrate employed in our simulations is a generic model of hydrophobic surfaces employed in previous simulation studies [80,81] and is characterized by a contact angle of 140° with a water droplet on the surface (as obtained in MD simulations) [81,82]. The substrate was made of 1600 atoms arranged in four equal layers of 400 atoms (67.48 Å × 67.48 Å) with a primitive cubic structure. The atoms of the substrate were maintained fixed during the whole simulation. Each atom was modeled as a Lennard–Jones sphere with σ = 3.374 Å and ϵ = 0.164 kcal/mol. The charge of each atom of the substrate was zero for the simulations with a neutral surface and −0.017763e for the simulations of a negatively-charged surface (which generates a surface electric field equivalent to a charge density of σ=−10 μC/cm^2^ = −0.62 e/nm^2^) to simulate the charge of the anionic latex used in the adsorption study.

#### 3.2.2. MD Simulations of Proteins in Implicit Water

We have performed molecular dynamics (MD) simulations of the proteins of interest using the GBIS model described above as implemented in NAMD Version 2.9 [83]. Newton’s equations of motion were solved with a time step of 2 fs, and electrostatic interactions were updated with a 4-fs time step (remember here that these are nominal times that, as discussed in Section 3.2.1, correspond to between 2 and 20 faster real experimental times due to the use of an implicit solvent model). The temperature was maintained constant at 298 K using the Langevin thermostat with a relaxation constant of 1 ${\mathrm{ps}}^{-1}$. All bonds between heavy atoms and hydrogen atoms were maintained rigid. The nonbonding Lennard–Jones interactions were cut off at a distance of 1.4 nm employing a switching function starting at 1.3 nm. All parameters for the GBIS energy calculation were set at their NAMD default values [77].

We used different tools available in the VMD software [29] to analyze the results during the simulations. Changes in the protein structure at the atomic coordinates level were characterized by the root mean square distance (RMSD) between the instantaneous atomic coordinates of the protein and their initial values:

(7) $$RMSD\left(t\right)=\sqrt{\frac{1}{N}\sum_{i=1}^{N}{\left[{\vec{r}}_{i}\left(t\right)-{\vec{r}}_{i}\left(0\right)\right]}^{2}},$$

where *N* is the number of atoms. Before applying Equation (7), the instantaneous protein structures are aligned with the initial structure so that rotations and translations of the whole protein do not contribute to the RMSD. We have also calculated the solvent-accessible surface area (SASA) of each protein, using a standard algorithm as implemented in VMD (see [29]). We also employed the “timeline” option in VMD to monitor the evolution of the secondary structure of the proteins during the simulations, computing the amount of α-helix and β-barrel conformations as a function of simulation time. Using the built-in VMD option, we have also calculated the inertia tensor of the proteins, the three principal moments of inertia of the protein ($I_{1}$, $I_{2}$ and $I_{3}$) and determined the three principal axis of inertia of the proteins. The size and shape of the proteins has been characterized as proposed in [30] by calculating the radius of gyration ($R_{g}$), the asphericity parameter (Δ) and the shape parameter (*S*). The radius of gyration $R_{g}$ is defined by:

(8) $$R_{g}^{2}\left(t\right)=\frac{\sum_{i=1}^{N}m_{i}{\left({\vec{r}}_{i}\left(t\right)-{\vec{r}}_{CM}\left(t\right)\right)}^{2}}{\sum_{i=1}^{N}m_{i}},$$

where ${\vec{r}}_{CM}$ is the center of mass of the protein. $R_{g}$ can be decomposed into three components $R_{1}$, $R_{2}$ and $R_{3}$, obtained by restricting the calculation in Equation (8) to each of the three principal axis of inertia. These components are related to $R_{g}$ by:

(9) $$R_{g}^{2}=R_{1}^{2}+R_{2}^{2}+R_{3}^{2}.$$

The asphericity parameter Δ characterizes the average deviation of the protein conformation from a spherical symmetry, and it is calculated from the three principal moments of inertia of the protein as:

(10) $$\Delta =\frac{3}{2}\frac{\sum_{i=1}^{N}{\left(I_{i}-I_{m}\right)}^{2}}{{\left(I_{1}+I_{2}+I_{3}\right)}^{2}},$$

where $I_{m}=\left(I_{1}+I_{2}+I_{3}\right)/3$. A sphere corresponds to Δ=0, and a rod has Δ=1. The shape parameter *S* is defined by:

(11) $$S=27\;\frac{\prod_{i=1}^{3}\left(I_{i}-I_{m}\right)}{I_{m}^{3}}.$$

S=0 corresponds to a sphere; S<0 corresponds to an oblate conformation; and *S* > 0 corresponds to a prolate conformation.

The protocols for the simulations were as follows. The three atomistic models of the proteins described in Section 3.2.1 were equilibrated in implicit water at 25 °C and neutral pH. After initial energy minimization, MD simulation runs were performed for each protein, until the computed structural magnitudes (RMSD and SASA) attain a stationary value. The full time evolution of these magnitudes is shown in the Supporting information.

The structures obtained from these simulations were employed as starting configurations for the calculations of the protein charge as a function of pH and the theoretical prediction of their isoelectric point (pI). The calculations were done using PropKa semi-empirical method Version 3.0 [53,54,55]. The PropKa calculations can be done directly from the crystallographic structures of the proteins (if available), but it is more appropriate to use structures corresponding to proteins equilibrated at the temperature of interest.

#### 3.2.3. MD Simulations of β-Casein Adsorption

We have performed 5 different simulations of β-casein adsorption.

In Simulation 1, we have considered the adsorption of a single protein onto a neutral surface. We started with the final protein coordinates obtained in the simulations described in Section 3.2.2. Initially, we placed the protein at a distance of 0.445 nm from the surface (as measured between the closest atom of the protein to the top of the surface). We placed the protein oriented in such a way that the most hydrophobic region (residues: 70, 73, 75–82, 85 and 126) is exposed towards the hydrophobic surface. This initial configuration was chosen in order to speed up simulations and save computational time.

In Simulation 2, we have performed the same simulation as in Simulation 1, but using a negatively-charged surface with σ=−0.62 e/nm^2^ as described in Section 3.2.1.

In Simulation 3, we studied the effect of pH from 7–4 onto an adsorbed protein at the negatively-charged surface. We started from the final configuration of Simulation 2, and we modified the protein structure by changing the protonation state of the amino acids to those corresponding to pH 4, as determined by the PropKa calculations described before. This change in pH affects the protonation state of 14 amino acids and the charge of the molecule changes from −8e–+6e.

In Simulations 4 and 5, we considered the effect of the coverage of the surface at the negatively-charged surface and pH 7. In Simulation 4, we started from the final configuration of Simulation 2 with a protein adsorbed to the surface, and we added another protein to the system (with initial distance and orientation as in Simulations 1 and 2). In Simulation 5, we started from the final configuration of Simulation 4, and we added a third protein to the system.

During all of these simulations, magnitudes such as total energy, RMSD, $R_{g}$ and the percentage of α-helix and β-barrel were monitored (see Table S2). The simulations were performed until these magnitudes were considered stable, as shown by the data provided in the Supplementary Information. As mentioned before, the evolution of these quantities is much faster in our implicit solvent simulations than in typical explicit solvent MD simulations.

Density profiles of adsorbed proteins were obtained using the density profile tool developed in [84].

## 4. Conclusions

This article describes simulation and experimental results of protein adsorption onto hydrophobic surfaces. Proteins of biotechnological interest, β-casein and β-lactoglobulin, as well as BSA are considered. MD simulations allowed us to observe protein adsorption at the microscopic level. From our simulations with β-casein and previous studies with BSA and β-lactoglobulin, [15,27,31,85,86], we can conclude that for the case of hydrophobic surfaces, protein adsorption is mostly mediated by the hydrophobic effect and to a lesser extent by electrostatic interactions. We found that more hydrophobic residues of proteins are oriented toward this kind of sorbent.

The combination of MD simulations and QCM was shown to be a powerful tool to understand the role of protein-protein interaction in the adsorption process that affects the protein orientation and the available surface for adsorption per protein molecule. In addition, protein-protein interactions may induce re-arrangements in protein structure modifying the thickness of the adsorbed film. In this way, we have obtained rigid monolayers in the case of the more globular proteins (BSA and β-lactoglobulin) and multilayers for the most hydrophobic and flexible protein studied (β-casein).

Finally, we have inquired into the behavior of protein-coated latex microparticles. We have observed that when a high coverage is achieved, the pI of the complex is very close to the pI of the protein in solution. We believe that the charge of the latex is reduced because of the low permittivity of the medium (protein) in comparison with water. Therefore, the charge of the latex only has a minor effect on pI. On the other hand, we have attributed the ion condensation to the lower-than-expected charge densities of the protein complexes.

## Figures and Tables

**Table 1:** Summary of protein data and results obtained from MD simulations of proteins in implicit solvent at 298 K (see the text for details). The RMSD corresponds to the value of the final equilibrium state compared with the initial states. The SASA is given both for the initial (${\mathrm{SASA}}_{i}$) and equilibrium (${\mathrm{SASA}}_{eq}$) states. The values for the asphericity parameter Δ, shape parameter *S*, radius of gyration $R_{g}$ and components of the radius of gyration ($R_{1}$, $R_{2}$, $R_{3}$) correspond to averages computed in the equilibrium state. These parameters are calculated using Equations (7)–(11). The simulation pI corresponds to the PropKa calculation, and the experimental pI corresponds to the value provided by the suppliers. SASA, solvent-accessible surface area.

Protein	Number of Amino Acids	RMSD (nm)	${\mathrm{SASA}}_{i}$ (nm^2^)	${\mathrm{SASA}}_{\mathrm{eq}}$ (nm^2^)	$R_{g}$ (nm)	$R_{1}^{2}$ (nm^2^)	$R_{2}^{2}$ (nm^2^)	$R_{3}^{2}$ (nm^2^)	Δ	*S*	pI (Sim)	pI (Exp)
β-cas	195	1.0	118	124	2.73	3.2	3.0	1.3	6 × $10^{-2}$	−3 × $10^{-2}$	4.9	4.6–5.1
β-lg	162	0.3	87	96	2.14	1.6	1.5	1.4	8 × $10^{-4}$	−3 × $10^{-5}$	4.7	5.1
BSA	582	0.4	284	317	3.86	6.4	4.7	3.9	2 × $10^{-2}$	+4 × $10^{-3}$	5.5	5.3

**Table 2:** Properties of protein films over polystyrene (PS) surfaces obtained at the end of the isotherms or by direct adsorption of protein solutions (1-mg/mL concentration, T = 298 K, pH 6 for BSA and β-lactoglobulin and pH 7 for β-casein). The adsorbed mass $m_{f}$ is obtained by QCM-D, and the thickness $h_{\mathrm{AFM}}$ is obtained by AFM in air (see the height profiles in Figure 5). The area per protein is computed from $m_{f}$ of the direct adsorption using Equation (1).

Protein Film	Isotherm $m_{f}$ (mg/m^2^)	Direct ads $m_{f}$ (mg/m^2^)	$a_{p}$ (nm^2^)	$h_{\mathrm{AFM}}$ (nm)
PS-β-lg	1.41 ± 0.03	2.1 ± 0.2	14.4	∼3
PS-BSA	1.88 ± 0.08	3.8 ± 0.4	29	∼5
PS-β-cas	3.7 ± 0.1	6.1 ± 0.7	–	∼7

**Table 3:** Isoelectric point (pI) of the protein-latex complexes as obtained from the electrophoretic measurements reported in Figure 8. For comparison, we also include the pI obtained by simulation and the experimental pI of each protein reported in Table 1.

Protein	pI (Complex) Anionic Latex	pI (Complex) Cationic Latex	pI (Protein) Simulation	pI (Protein) Experimental
β-cas	4.9	5.7	4.9	4.6–5.1
β-lg	4.1	6.0	4.7	5.1
BSA	4.8	5.9	5.5	5.3

## Supplementary Materials

The following are available online at www.mdpi.com/1996-1944/10/8/893/s1, Figures S1: Charges (in units of electronic charge) for each protein molecule as a function of pH. The electrostatic charge of the proteins has been calculated from the 3D structures equilibrated in implicit water (PropKa calculation), Figure S2: Time evolution of the root mean squared deviation (RMSD) between the β-casein structure in solution and adsorbed onto a neutral hydrophobic surface or a negative hydrophobic surface, Figure S3: Time evolution of the rootmean squared deviation (RMSD) of the β-casein structure adsorbed onto a negative hydrophobic surface from pH 7 to pH 4, Figure S4: Time evolution of the solvent-accesible surface area (SASA) between the β-casein structure adsorbed onto a negative hydrophobic surface from pH 7 to pH 4, Table S1: Composition of buffered solutions, Table S2: Secondary structure (β-sheet and α-helix content) of β-casein protein obtained in MD simulations in bulk or adsorbed onto surfaces of different charge and different pH and Table S3: Values of critical charge density ($\sigma_{crit}$) using Manning model, eqn (11), and Bocquet et al. model, eqn (12), calculated for spherical colloids ($a_{anionic}$ = 80 nm) and ($a_{cationic}$ = 255 nm) immersed in different buffered solutions.

## Abbreviations

The following abbreviations are used in this manuscript:

MD	Molecular dynamics
AFM	Atomic force microscopy
QCM-D	Quartz crystal microbalance with dissipation monitoring
BSA	Bovine serum albumin
β-lg	β-lactoglobulin
β-cas	β-casein
