# Supplementary material for: Adsorption of Milk Proteins (β-Casein and β-Lactoglobulin) and BSA onto Hydrophobic Surfaces
Source: Materials (Basel). 2017 Aug 2;10(8):893. doi: 10.3390/ma10080893 (PMC5578259; doi:10.3390/ma10080893)
Supplement: Supplementary file 1 [file materials-10-00893-s001.pdf]

Electronic Supporting Information: Adsorption of milk proteins (β-casein and β-lactoglobulin) and BSA onto hydrophobic surfaces

Leonor Pérez-Fuentes[1,*], Carlos Drummond[2,3], Jordi Faraudo[4], and Delfi Bastos-González[1]

[1]Biocolloid and Fluid Physics Group, Department of Applied Physics, University of Granada, Av. Fuentenueva 2, E-18001 Granada, Spain.
[2]CNRS, Centre de Recherche Paul Pascal (CRPP), UPR 8641, F3300 Pessac, France, Université de Bordeaux, CRPP, UPR 8641, F-33600 Pessac, France.
[3]Université de Bordeaux, CRPP, UPR 8641, F-33600 Pessac, France.
[4]Institut de Ciència de Materials de Barcelona (ICMAB-CSIC), Campus de la UAB, E-08193 Bellaterra, Barcelona, Spain. E-mail: jfaraudo@icmab.es

Materials and Methods

In the experimental part, we used several buffered solutions. The details of the composition of the buffers are presented below:

Table S1. Composition of buffered solutions

pH	Component	Salt concentration (mM)	Ionic strength (mM)
4	AcH	13.50	2.01
5	AcH	3.15	2.00
6	NaH_2PO_4	1.79	2.00
7	NaH_2PO_4	1.13	2.01
8	H_3BO_3	15.00	0.82
9	H_3BO_3	5.14	1.90
10	H_3BO_3	2.20	1.98

All solutions were prepared with double distilled and deionized (Milli-Q) water. The desired pH was achieved by addition of few dropplets of a NaOH solution (this component is taken into account for the calculation of ionic strength). In the case of the buffer for preparing β-casein solution, we used 10 mM of Bis-Tris and the pH was adjusted with dilute HCl (2.40 mM of ionic strength).

Results

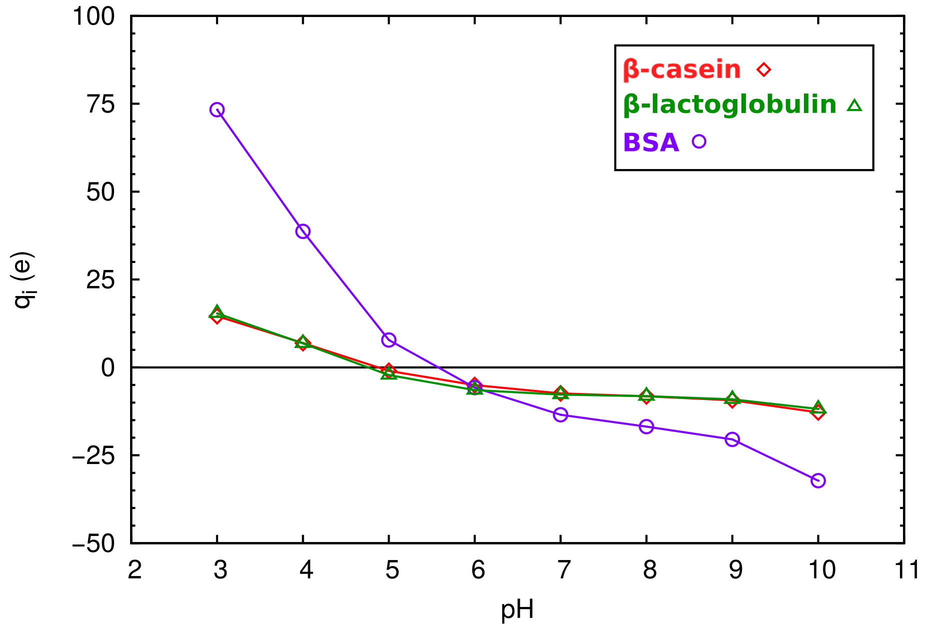

Figure S1. Charges (in units of electronic charge) for each protein molecule as a function of pH. The electrostatic charge of the proteins has been calculated from the 3D structures equilibrated in implicit water (PropKa calculation).

Table S2. Secondary structure (β-sheet and α-helix content) of β-casein protein obtained in MD simulations in bulk or adsorbed onto surfaces of different charge and different pH

Simulation	β-sheet	α-helix
Bulk (pH=7)	77%	8%
Neutral surface (pH=7)	40%	43%
Negative surface (pH=7)	37%	44%
Negative surface (pH=4)	36%	45%

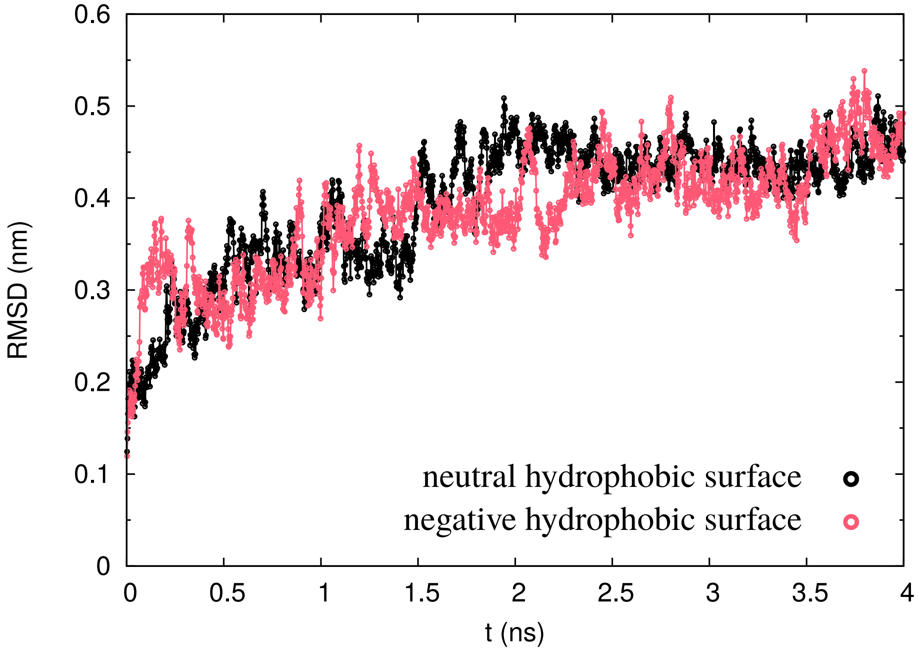

Figure S2. Time evolution of the root mean squared deviation (RMSD) between the β-casein structure in solution and adsorbed onto a neutral hydrophobic surface or a negative hydrophobic surface.

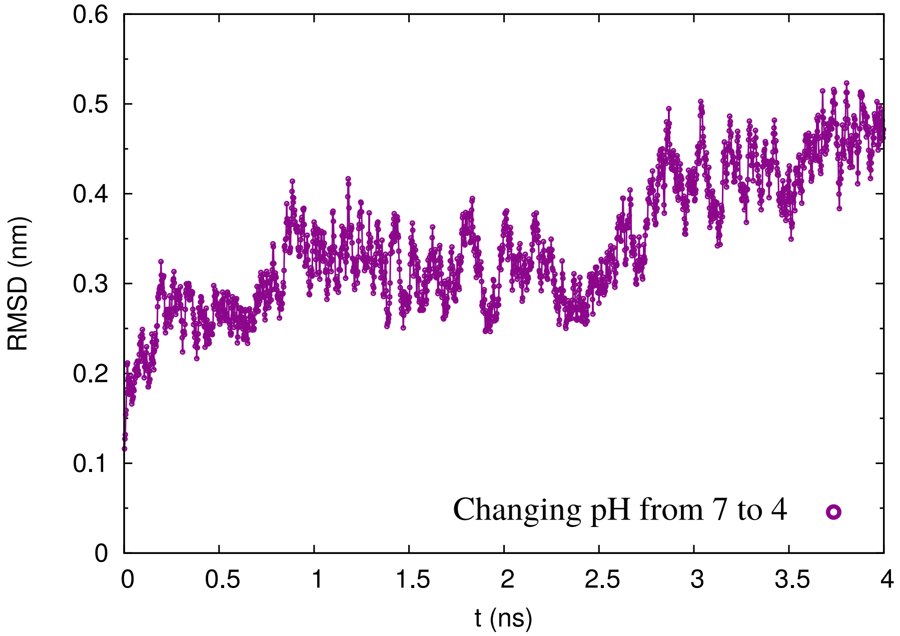

Figure S3. Time evolution of the root mean squared deviation (RMSD) of the β-casein structure adsorbed onto a negative hydrophobic surface from pH 7 to pH 4.

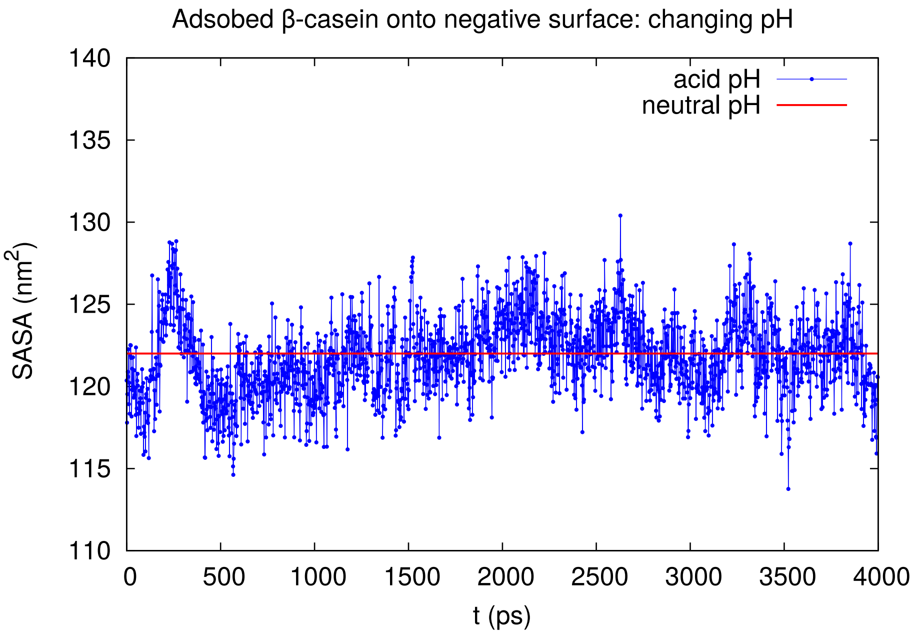

Figure S4. Time evolution of the solvent-accesible surface area (SASA) between the β-casein structure adsorbed onto a negative hydrophobic surface from pH 7 to pH 4.

Table S3. Values of critical charge density (σ_{crit}) using Manning model, eqn (11), and Bocquet *et al.* model, eqn (12), calculated for spherical colloids ($a_{anionic}$=80 nm) and ($a_{cationic}$=255 nm) immersed in different buffered solutions

pH	Ionic concentration (mM)	Anionic latex Manning $\|\sigma_{crit}\|$ (e/nm^2)	Anionic latex Bocquet *et al.* $\|\sigma_{crit}\|$ (e/nm^2)	Cationic latex Manning $\|\sigma_{crit}\|$ (e/nm^2)	Cationic latex Bocquet *et al.* $\|\sigma_{crit}\|$ (e/nm^2)
3	1.00	0.068	0.052	0.063	0.048
4	2.01	0.080	0.071	0.076	0.067
5	2.00	0.080	0.071	0.076	0.067
6	1.90	0.080	0.071	0.076	0.067
7	1.57	0.080	0.071	0.076	0.067
8	0.82	0.064	0.048	0.059	0.044
9	1.90	0.079	0.069	0.075	0.066
10	1.98	0.071	0.074	0.076	0.067
